# Radial Heterogeneity of Microstructure and Mechanical Properties in a Resin Sand-Cast Mg-Gd-Y-Zn-Zr Alloy

**DOI:** 10.3390/ma19204316

**Published:** 2026-10-13

**Authors:** Shuxian Shi, Haoling Ge, Wenbing Zou, Hongxian Shen, Haokun Lin, Yongjiang Huang, Lunyong Zhang, Jianfei Sun

**Affiliations:** 1 National Key Laboratory for Precision Hot Processing of Metals, Harbin Institute of Technology, Harbin 150001, China; ssx18854091620@163.com (S.S.);; 2 Xinjiang Technology (Jiangsu) Co., Ltd., Nantong 226100, China; 3 Shanghai Aerospace Precision Machinery Research Institute, Shanghai 201600, China

**Keywords:** Mg-Gd-Y-Zn-Zr magnesium alloy, low-pressure resin sand casting, microstructure, mechanical properties, heterogeneity

## Abstract

**Highlights:**

- A thick-walled as-cast Mg-RE alloy exhibits radial microstructural heterogeneity.
- Local thermal conditions may contribute to the observed grain-size and texture variations.
- Mechanical properties vary spatially; the edge region exhibits the highest strength.

**Abstract:**

This study examines the radial microstructure, texture, tensile properties, and fracture morphology of a 100 mm diameter as-cast Mg-9.5Gd-0.6Y-0.2Zn-0.35Zr (wt.%) alloy prepared by low-pressure resin sand casting. XRD identifies α-Mg as the main resolved phase, while SEM/EDS reveals Zr-rich and Gd/Y-rich particles. The characteristic grain sizes obtained from one EBSD map per region are 88.4, 97.4, and 81.4 μm for the center, near-center, and edge, respectively. Nanoscale features observed by TEM are provisionally associated with β″ (Mg_3_Gd); their phase identity and strengthening contribution remain unconfirmed. Among the sampled regions, the edge has the highest reported mean ultimate tensile strength (237.0 MPa) and estimated 0.2% offset stress (143.9 MPa). The fracture surfaces predominantly show cleavage-like features. These findings describe the sampled radial locations; local cooling histories and individual strengthening contributions were not quantified.

## 1. Introduction

Magnesium alloys have attracted considerable attention as highly promising lightweight structural materials in the aerospace, automotive, and 3C (computer, communication, and consumer electronics) industries, owing to their low-density, high specific strength, good damping capacity, and promising electromagnetic shielding performance [1,2,3,4,5].

Magnesium is the lightest structural engineering metal, with a density of approximately 1.74 g/cm^3^, which is significantly lower than that of aluminum (2.7 g/cm^3^), titanium (4.5 g/cm^3^), and iron (7.9 g/cm^3^) [6,7,8]. In addition, magnesium and its alloys are considered resource-abundant and can be highly recycled, making them attractive candidates for sustainable structural applications [6,9,10]. However, their relatively poor corrosion resistance and limited absolute strength remain key challenges restricting broader industrial adoption [6].

Rare-earth (RE) alloying is an effective strategy for tailoring the microstructure and enhancing the comprehensive mechanical properties of magnesium alloys by promoting solid-solution strengthening, precipitation strengthening, and grain refinement [4,11,12,13]. The strong interaction between RE atoms and the Mg matrix can effectively improve strength while maintaining acceptable ductility, making RE-containing Mg alloys an important research direction for high-performance lightweight materials [3,13,14].

Zr is widely recognized as a highly effective grain refiner for non-aluminum-bearing magnesium alloys, capable of significantly refining the as-cast grain size and thus improving the overall mechanical response [15]. Adding gadolinium (Gd) to magnesium alloys effectively enhances mechanical strength through multiple synergistic effects, including solid solution strengthening caused by Gd-induced lattice distortion [13], precipitation strengthening from nanoscale Mg-Gd metastable phases during aging treatment [16], and grain refinement strengthening [17]. The introduction of Zn in Mg-RE alloys can influence solid-solution behavior and phase stability [18]. In the present alloy system, due to the relatively low Zn content, the formation of long-period stacking ordered (LPSO) structures is not expected [19]. Instead, Zn atoms can partially substitute Mg atoms in the lattice [20] because of their smaller atomic radius (0.135 nm for Zn compared with 0.160 nm for Mg), leading to local lattice contraction [21,22]. Together with the lattice expansion induced by larger rare-earth elements such as Gd and Y, the size mismatch between Zn and Mg atoms contributes to lattice distortion strengthening and overall solid-solution strengthening [23,24,25].

In this work, a nominal Mg-9.5Gd-0.6Y-0.2Zn-0.35Zr (wt.%) alloy was prepared by low-pressure sand casting under argon protection. We compare the microstructure, crystallographic texture, mechanical properties, and fracture morphology at three radial locations in one as-cast cylinder. The contribution is a location-resolved description of a thick-walled casting made by this process.

## 2. Materials and Methods

### 2.1. Alloy Preparation

**Figure 1:**
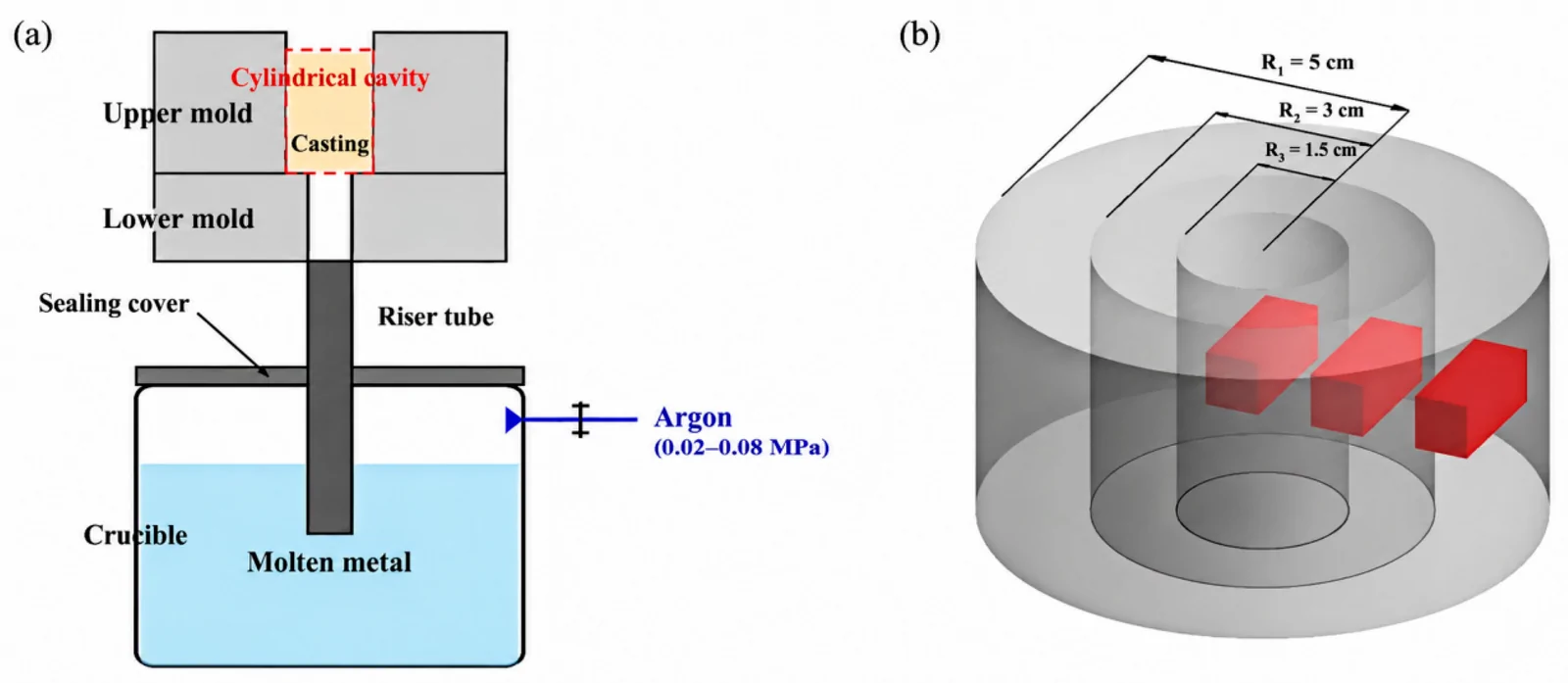
(**a**) Schematic diagram of the low-pressure sand casting apparatus for Mg alloy ingot preparation; (**b**) the three radial sampling zones.

The material used in this study is an Mg-Gd-Y-Zn-Zr rare-earth magnesium alloy with a nominal composition of Mg-9.5Gd-0.6Y-0.2Zn-0.35Zr (*wt*.%). To investigate the as-cast microstructure, the alloy was prepared using a low-pressure sand casting method [26,27]. The experimental setup is shown in Figure 1a, which illustrates the low-pressure sand casting apparatus used for magnesium alloy ingot preparation. The stated composition is nominal; the bulk chemical composition of this casting was not measured.

The system consists of a sealed crucible furnace, a ceramic riser tube, and a split mold. Under argon protection, the melt filled the mold from the bottom through the riser tube. The pressure range shown in Figure 1a is schematic; the actual pressure–time profile was not recorded. The cylindrical casting had a diameter of 100 mm. All microstructural and mechanical specimens used in this study were sampled from this casting. The center, near-center, and edge sampling zones were located at radial distances of 0–1.5, 1.5–3, and 3–5 cm, respectively. Local cooling rates were not measured.

### 2.2. Microstructural Characterization

The phase constitution was examined by X-ray diffraction (XRD, Panalytical X’Pert PRO) with Cu-Kα radiation over 2θ = 10–90° at 12°/min. Optical microscopy (OM, VHX-2000E, KEYENCE Corporation, Osaka, Japan) and scanning electron microscopy (SEM, Thermo Fisher Scios 2, Thermo Fisher Scientific, Waltham, MA, USA) with energy-dispersive X-ray spectroscopy (EDS, Oxford Instruments Xplore 30, Oxford Instruments NanoAnalysis, High Wycombe, UK) were used for microstructural observation and elemental mapping. Following preliminary observation of similar grain sizes within each zone, one representative EBSD field was selected in each radial zone; the step size was 3.8 μm, with acquisition grids of 561 × 417–421 points (approximately 2.13 × 1.58–1.60 mm); acquisition took about 10 min per map, and the maps were automatically cleaned in AZtecCrystal (version 2.1, Oxford Instruments NanoAnalysis, High Wycombe, UK). TEM was performed on mechanically ground and low-energy ion-milled foils (Gatan PIPS II) with a TALOS F200X G2(Thermo Fisher Scientific, Waltham, MA, USA) at 200 kV.

### 2.3. Mechanical Testing

**Figure 2:**
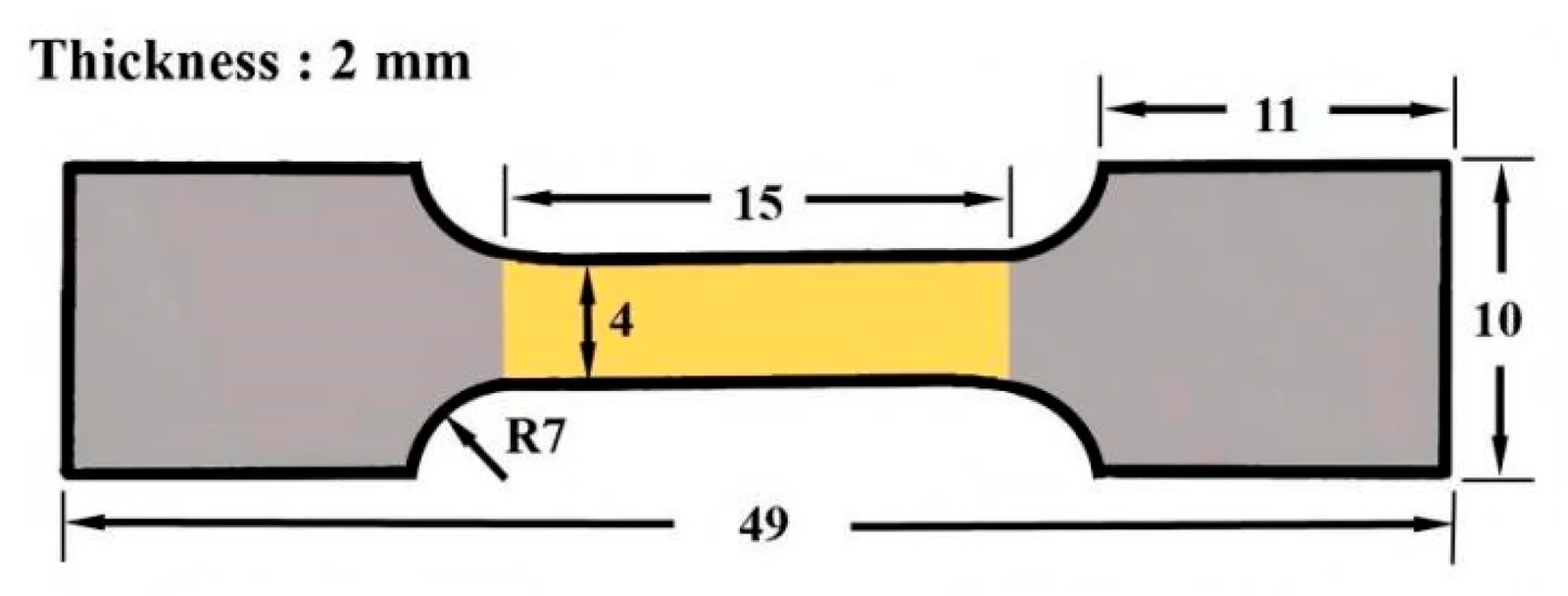
Schematic illustration of the geometric dimensions of the tensile specimen.

Room-temperature tensile tests were performed with reference to GB/T 228.1-2021 [28] using an Instron 3382 (Instron, Norwood, MA, USA) at a crosshead speed of 0.5 mm/min. All tensile specimens were taken from the same casting in the center, near-center, and edge zones and loaded parallel to the cylinder axis. The 15 mm dimension in Figure 2 is the parallel-section length. No extensometer was used; the reported Rp0.2 values are estimates derived from crosshead displacement.

Five tensile specimens were tested at each sampling position; results are reported as mean ± standard deviation. The detailed geometric dimensions of the tensile specimens are schematically illustrated in Figure 2. Vickers hardness (HV) was measured using a Vickers microhardness tester (Wolpert Wilson 6100HVT-1000,Wolpert Wilson Instruments, Norwood, MA, USA) with an applied load of 500 g (4.9 N) and a dwell time of 10 s. To ensure data reliability, at least eight indentation points were randomly selected for each sample with a spacing of at least three times the diagonal length between adjacent indentations to avoid interaction effects, and mean and standard deviation were calculated.

## 3. Results and Discussion

### 3.1. Microstructure and Phase Constituents

**Figure 3:**
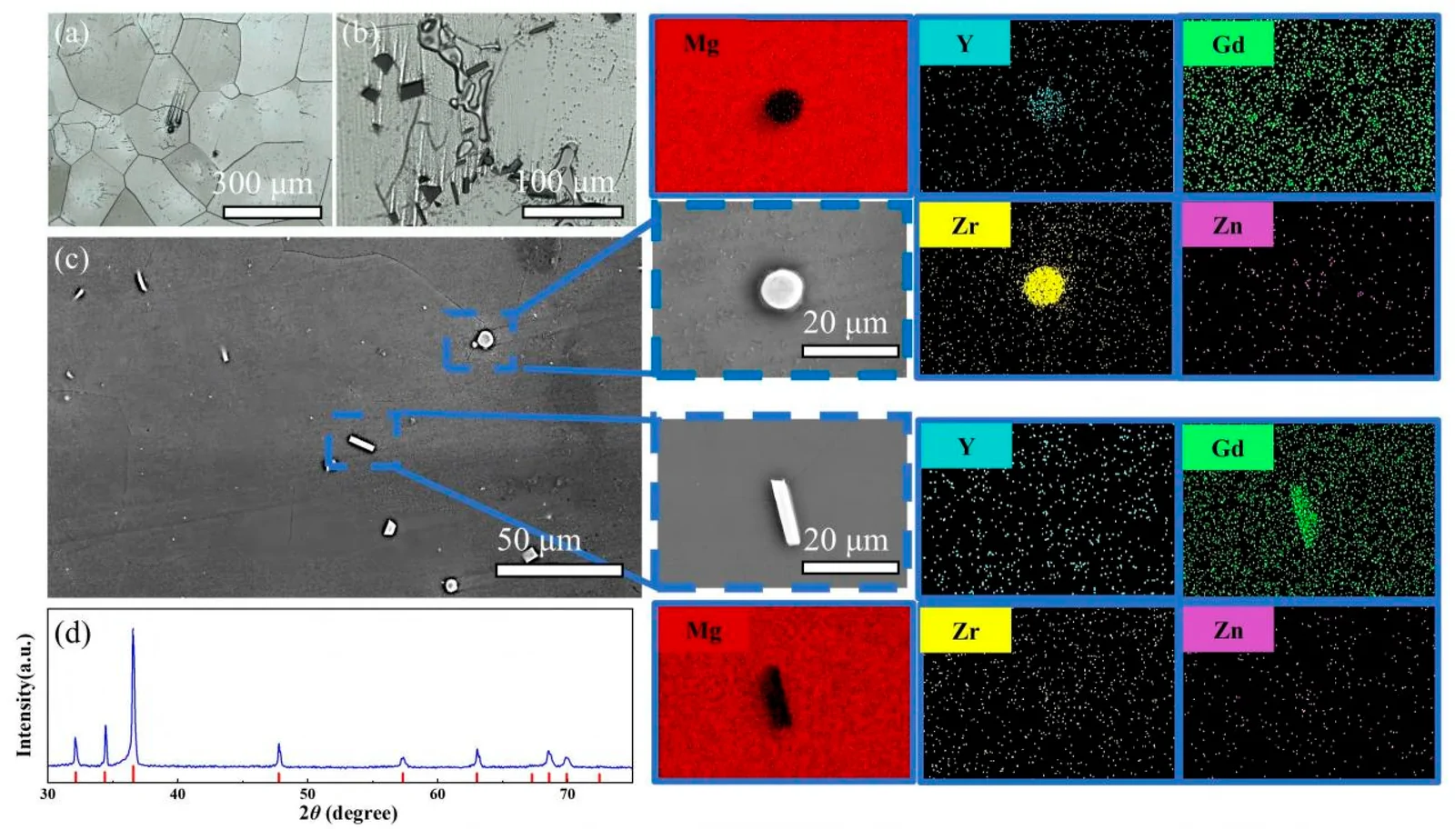
Microstructure and elemental distribution in the center region: (**a**,**b**) optical micrographs; (**c**) SEM image and corresponding EDS maps; (**d**) XRD pattern.

The representative macrostructure and basic microstructural features of the as-cast alloy in the center region are presented in Figure 3a–c. The optical micrographs show a typical α-Mg matrix with a relatively uniform equiaxed grain structure. In addition to the matrix, two distinct types of second-phase particles can be clearly identified.

As shown in Figure 3b,c, SEM/EDS reveals fine Zr-rich particles and blocky particles enriched in Gd and Y in the examined field; Zn is also locally detected. Their elemental enrichment does not establish precise phase stoichiometry or a specific nucleation mechanism.

The Zr-rich and Gd/Y-rich particles appear on the micrometer scale in the examined images. No clear diffraction peaks from these particles are resolved in the XRD pattern (Figure 3d), which is dominated by α-Mg.

This indicates that the alloy in the as-cast state is mainly composed of an α-Mg matrix, while the second phases exist in the form of fine Zr-rich and Gd/Y-rich particles with limited detectability in bulk diffraction characterization.

Zr-rich particles may be relevant to grain refinement, although their role as nucleation sites and their strengthening contribution were not directly quantified. Blocky Gd/Y-rich particles are observed to be dispersed within the matrix and along grain boundaries. Their volume fraction and contribution to deformation were not measured quantitatively.

### 3.2. EBSD Analysis

**Figure 4:**
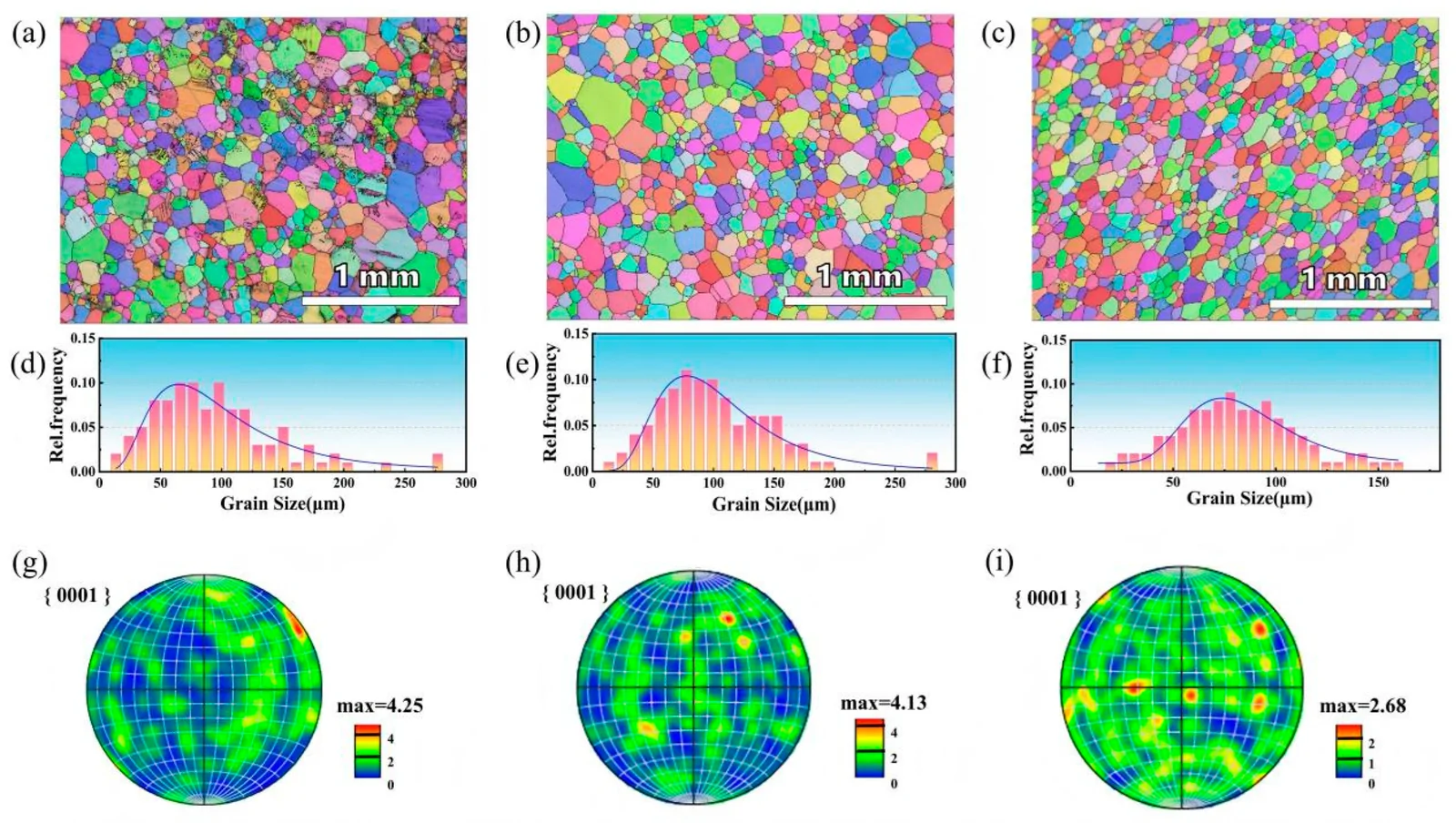
EBSD inverse pole figure (IPF) maps (**a**–**c**), grain size distributions (**d**–**f**), and corresponding {0001} pole figures (**g**–**i**) of the as-cast Mg-9.5Gd-0.6Y-0.2Zn-0.35Zr alloy cylinder for the center, near-center, and edge regions, respectively.

Figure 4 shows one EBSD map from each radial zone. The characteristic grain sizes obtained by log-normal fitting are 88.4, 97.4, and 81.4 μm for the center, near-center, and edge fields, respectively. These single-field values describe the sampled areas; within-zone variability and statistical significance were not determined. In particular, the center value is below the near-center value, so the three values do not show a monotonic radial trend. The {0001} pole figures differ visually, but the texture-intensity units and their orientation relative to the tensile axis could not be verified from the retained records. Differences in local cooling or solute distribution remain possible explanations rather than measured causes.

The EBSD maps demonstrate differences among the examined fields; spatial variation within each zone was not quantified.

### 3.3. TEM Characterization

**Figure 5:**
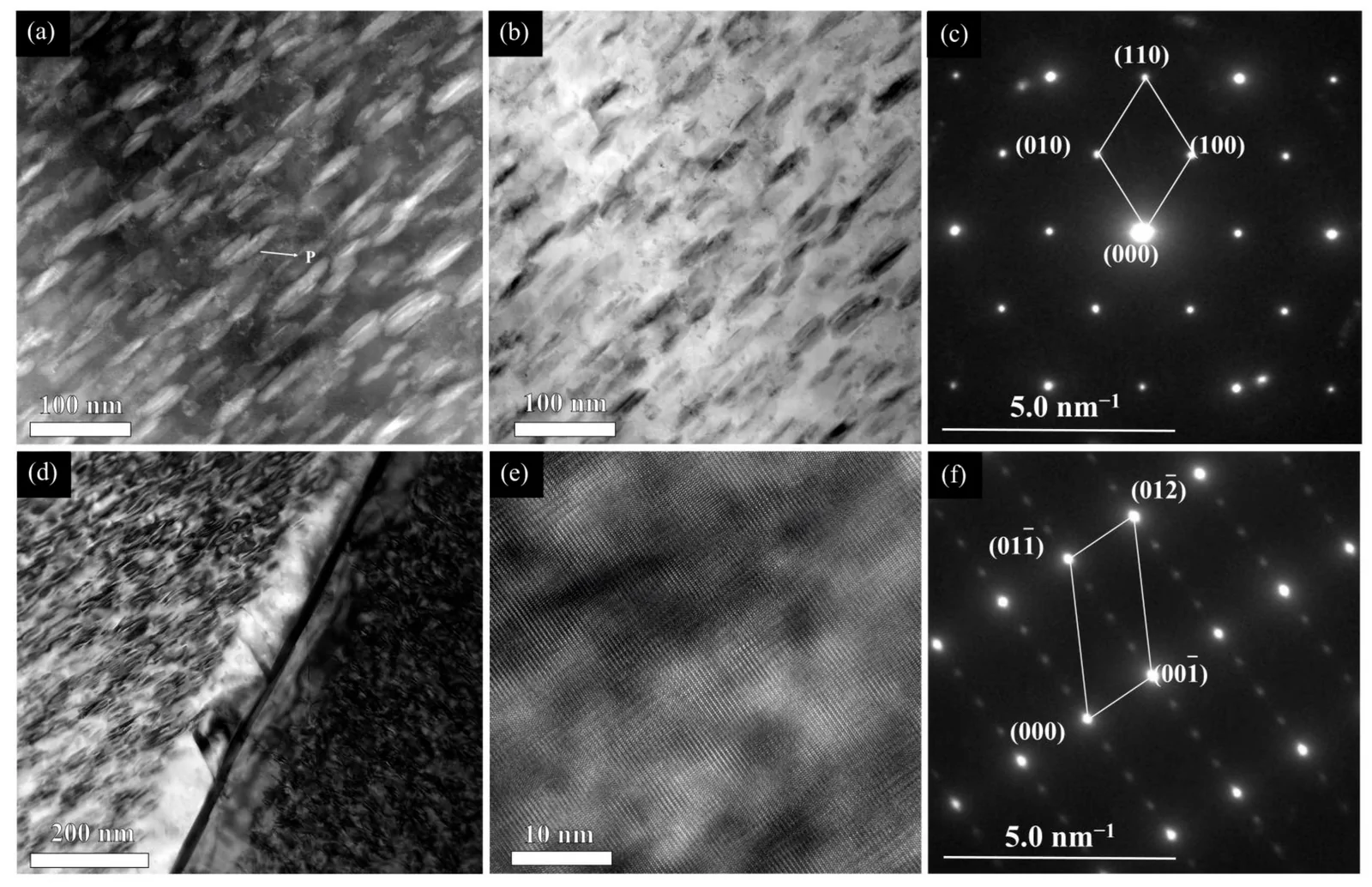
TEM observations of the as-cast center region: (**a**,**b**) images showing nanoscale contrast features (P marks a representative feature in (**a**)); (**c**) SAED pattern with annotated reflections, used for a provisional assignment to β″ (Mg_3_Gd); (**d**) nanoscale contrast features near a grain boundary; (**e**) HRTEM image showing lattice contrast; and (**f**) SAED pattern assigned to the α-Mg matrix.

TEM revealed nanoscale contrast features in the as-cast specimen shown in Figure 5. The TEM specimen was sampled from the center region of the casting. These observations describe the center-zone specimen and are not used as evidence of a radial TEM trend across all three zones.

Nanoscale features are visible in Figure 5a,b. The SAED pattern in Figure 5c is provisionally consistent with β″ (Mg_3_Gd), but the diffraction geometry and local composition needed for definitive phase identification were not established. The observations show features in the as-cast state, without determining whether they formed during solidification or subsequent cooling.

Nanoscale features and dislocation contrast are visible near a grain boundary in Figure 5d. Their proximity alone does not establish a particle–dislocation interaction or a measurable contribution to strength.

The HRTEM image in Figure 5e shows local lattice contrast, and Figure 5f shows an SAED pattern assigned to the α-Mg matrix. No phase-specific lattice-spacing, FFT, or local-composition analysis is presented for the nanoscale features; these images therefore do not independently confirm β″ or quantify precipitation strengthening.

Several limitations should be noted. The *β*″ (Mg_3_Gd) phase was only tentatively identified by SAED and requires further crystallographic and chemical confirmation. Cooling rates during casting were not directly measured, and this study is limited to the as-cast condition without exploring age hardening. The individual contributions of grain size, texture, and precipitation to property variations could not be quantitatively separated.

### 3.4. Mechanical Properties

**Figure 6:**
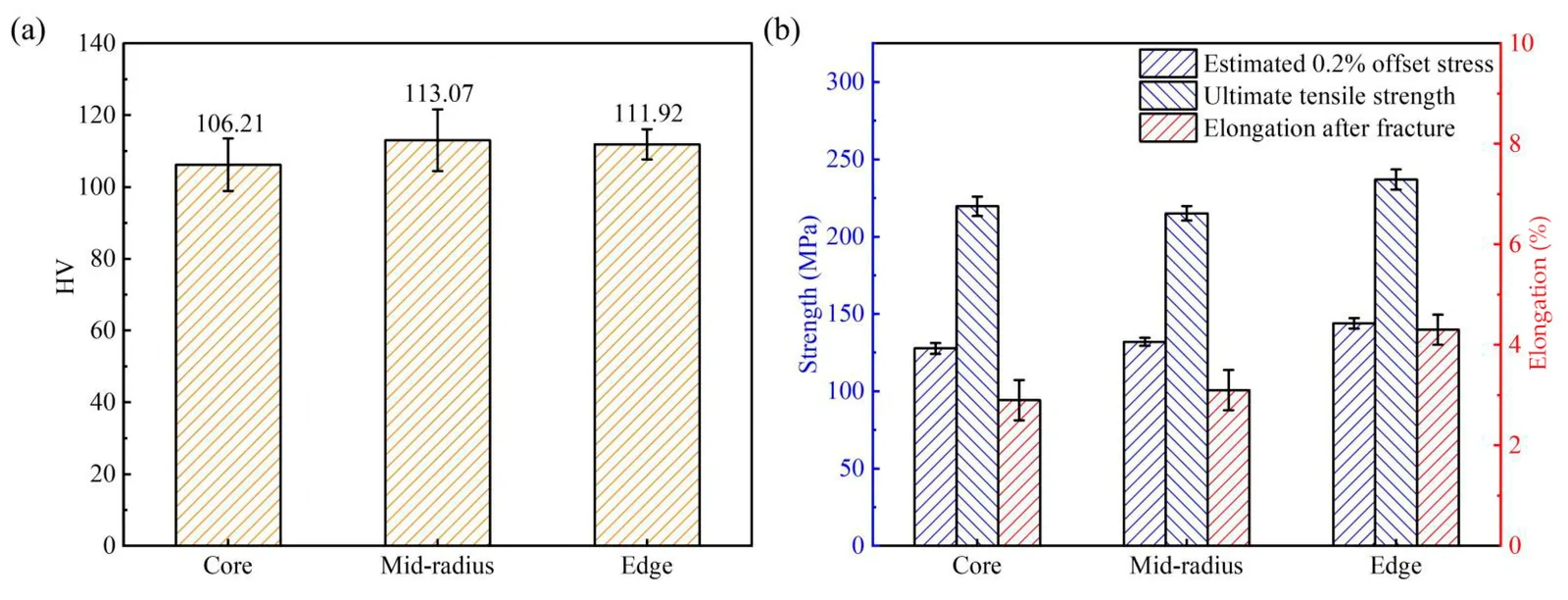
Mechanical properties of the Mg-9.5Gd-0.6Y-0.2Zn-0.35Zr rare-earth magnesium alloy sampled from the center (Core), near-center (Mid-radius), and edge (Edge) regions: (**a**) average Vickers microhardness values with error bars; (**b**) tensile properties, including estimated 0.2% offset stress (labeled “Yield strength” in the artwork), ultimate tensile strength, and elongation to failure. Error bars denote standard deviations.

Following the systematic evaluations of the microstructure, crystallographic texture, and nanoscale features in the preceding sections, the macroscopic mechanical responses of the Mg-9.5Gd-0.6Y-0.2Zn-0.35Zr alloy were investigated to establish a regional comparison across different spatial regions. The room-temperature tensile properties and Vickers microhardness values acquired from the center, near-center, and edge regions of the cast cylinder are presented in Figure 6. The average Vickers microhardness values across the three regions are presented in Figure 6a, with error bars representing standard deviations. The average tensile properties, including estimated 0.2% offset stress, ultimate tensile strength, and elongation to failure, are summarized in Figure 6b.

The detailed values of estimated 0.2% offset stress (Rp0.2), ultimate tensile strength (UTS), elongation to failure, and average Vickers hardness are systematically summarized in Table 1.

Specifically, the center region has a reported mean estimated Rp0.2 of 127.0 ± 3.5 MPa, ultimate tensile strength (UTS) of 219.7 ± 6.3 MPa, and elongation to failure of 2.9 ± 0.4%. The near-center values are 132.0 ± 2.6 MPa, 215.1 ± 4.7 MPa, and 3.1 ± 0.4%, respectively. The edge values are 143.9 ± 3.3 MPa, 237.0 ± 6.5 MPa, and 4.3 ± 0.3%, respectively.

The spatial variation in the Vickers hardness across the cylindrical ingot is shown in Figure 6a. The average microhardness of the center region is determined to be 106.2 ± 7.3 HV. Correspondingly, the average hardness values for the near-center and edge regions are 113.0 ± 8.6 HV and 111.9 ± 4.2 HV, respectively.

The edge field has the smallest reported characteristic grain size (81.4 μm), and the edge specimens have the highest reported mean tensile strengths. This association is qualitatively consistent with a grain-size contribution [29]. However, the center field is finer than the near-center field while its reported mean Rp0.2 is lower, and the near-center zone has the highest mean hardness. The three fields therefore do not establish a monotonic Hall–Petch relation or the relative contributions of grain size, texture, particles, and solute content.

To further contextualize the mechanical performance of the present alloy, a comparison with reported as-cast Mg–Gd–Y–Zn–Zr-based alloys prepared by industrial-scale casting processes is summarized. Xu et al. [30] fabricated an Mg-8.2Gd-3.8Y-1.0Zn-0.4Zr alloy by semi-continuous casting into a large ingot (*φ*280 mm) and reported a clear radial gradient: the center region exhibited a UTS of 187 MPa, tensile yield strength (TYS) of 119 MPa, and elongation of 2.1%, whereas the surface region reached a UTS of 241 MPa, TYS of 156 MPa, and elongation of 6.0%. Xia and Yang [31] prepared semi-continuously cast Mg-8.5Gd-4.5Y-*x*Zn-0.4Zr alloy ingots (*φ*162 mm); the Zn-free alloy achieved the highest strength (UTS 229 MPa, YS 185 MPa), whereas the 3.3 wt.% Zn alloy exhibited the best elongation (4.5%). Li et al. [32] cast an Mg-7Gd-3Y-1Nd-1Zn-0.5Zr alloy into a preheated permanent low-carbon steel mold and obtained a UTS of 187 MPa, YS of 145 MPa, and elongation of 3.1%. In comparison, the present as-cast Mg-9.5Gd-0.6Y-0.2Zn-0.35Zr alloy, fabricated by low-pressure sand casting (φ100 mm), achieves a UTS ranging from 219.7 to 237.0 MPa across the three regions. Notably, the UTS of the present center region (219.7 MPa) is substantially higher than that of the semi-continuously cast counterpart at the center (187 MPa) reported by Xu et al. [30]. The reported characteristic grain-size comparison is descriptive (88.4 μm vs. 126 μm), while the alloy compositions and routes differ. The reported 0.2% offset stress here (127.0–143.9 MPa) is below the comparison value at the surface (156 MPa) [30] and the Mg-8.5Gd-4.5Y-based alloy (185 MPa) [31]. The nominal Y contents differ (0.6 *wt*.% vs. 3.8–4.5 *wt*.%). These comparisons do not isolate composition, grain size, or casting-route effects. The elongation of the present alloy (2.9–4.3%) falls within the range reported for these as-cast RE-containing magnesium alloys [30,31,32]. Regarding hardness, the present alloy exhibits Vickers hardness values of 106.2–113.0 HV across the three regions (Table 1). A direct comparison with the literature is not attempted here, since hardness measurements are predominantly reported for aged rather than as-cast Mg-Gd-Y-Zn-Zr alloys [30,31,32]. These reported values provide context rather than a controlled process comparison.

The mechanical results vary among the sampled radial locations, as do the observed microstructural fields. Faster heat extraction near the mold wall is a plausible explanation for some differences, but local thermal histories were not measured. The data do not separate the effects of grain size, texture, solute content, and nanoscale features on strength.

The measured elongation to failure ranges from 2.9% to 4.3%. Limited room-temperature slip in the hexagonal close-packed α-Mg matrix may contribute to this modest ductility [33].

The present data do not quantify the separate strengthening contributions of grain size, solid solution, texture, or second phases.

### 3.5. Fracture Behavior

Fracture surfaces from tensile specimens in the three radial zones were examined to compare the observed fracture morphologies.

**Figure 7:**
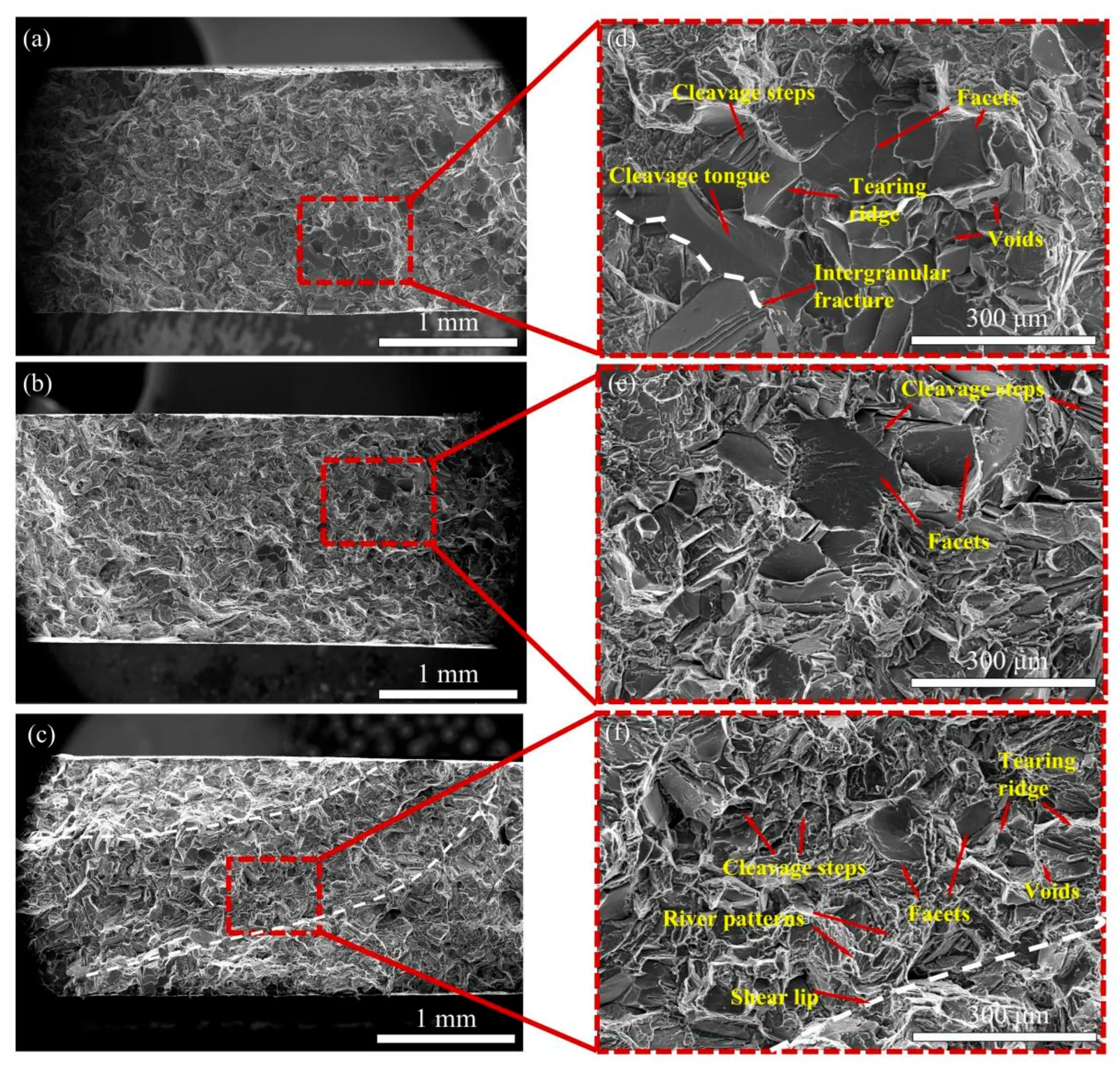
SEM micrographs showing the tensile fracture surface morphologies of the as-cast Mg-9.5Gd-0.6Y-0.2Zn-0.35Zr alloy at different spatial locations: (**a**–**c**) low-magnification overviews of the center, near-center, and edge regions, respectively; (**d**–**f**) corresponding high-magnification images showing characteristic fracture features. Red arrows indicate the labeled fracture features, and white dashed lines delineate selected regions of the fracture surfaces.

The tensile fracture surfaces are shown in Figure 7. At low magnification (Figure 7a–c), cleavage-like features occur in all three regions. A local area of larger facets is visible in the center specimen (Figure 7a), but the fracture image alone cannot establish a coarse-grained aggregation zone.

At higher magnification, the center specimen (Figure 7d) shows cleavage-like steps, tearing ridges, facets, and isolated microvoids. Tongue-like markings may reflect local deformation, but their origin in crack–twin interactions is not established by fractography alone. The near-center specimen (Figure 7b,e) also has relatively large facets, whereas the edge specimen (Figure 7c,f) has smaller visible facets and a localized shear-lip-like region. The images do not establish a one-to-one relationship between facet size, the single-field EBSD grain sizes, and local cooling rate.

Secondary cracks are visible near some facets. Although particles and phase boundaries might influence crack paths, the available fracture images do not directly identify crack-initiation sites or establish a dual strengthening and embrittlement role for the RE-rich particles.

## 4. Conclusions

The examined locations in the 100 mm diameter, nominal Mg-9.5Gd-0.6Y-0.2Zn-0.35Zr casting show radial differences in the observed microstructure and reported mechanical properties. XRD resolves α-Mg as the main phase, while SEM/EDS shows Zr-rich and Gd/Y-rich particles without determining their exact phase identities. The characteristic grain sizes from one EBSD field per region are 88.4, 97.4, and 81.4 μm for the center, near-center, and edge. The edge has the highest reported mean ultimate tensile strength (237.0 MPa) and an estimated 0.2% offset stress (143.9 MPa).

Nanoscale features were observed in an as-cast TEM specimen, but their provisional assignment to β″ (Mg_3_Gd), formation stage, and contribution to strengthening require further evidence. The examined fracture surfaces predominantly show cleavage-like or quasi-cleavage features. Possible effects of radial heterogeneity on service performance remain to be tested.

## Figures and Tables

**Table 1:** Room-temperature tensile properties and Vickers hardness of the as-cast Mg-9.5Gd-0.6Y-0.2Zn-0.35Zr alloy at different spatial locations (mean ± standard deviation).

Spatial Location	Estimated 0.2% Offset Stress (Rp0.2/MPa)	Ultimate Tensile Strength (Rm/MPa)	Elongation (A/%)	Vickers Hardness (HV)
Center	127.0 ± 3.5	219.7 ± 6.3	2.9 ± 0.4	106.2 ± 7.3
Near-center	132.0 ± 2.6	215.1 ± 4.7	3.1 ± 0.4	113.0 ± 8.6
Edge	143.9 ± 3.3	237.0 ± 6.5	4.3 ± 0.3	111.9 ± 4.2

## Data Availability

The data presented in this study are available on request from the corresponding author. The data are not publicly available due to privacy or ethical restrictions.
